# Efficacy of EGFR-TKI Plus Chemotherapy or Monotherapy as First-Line Treatment for Advanced EGFR-Mutant Lung Adenocarcinoma Patients With Co-Mutations

**DOI:** 10.3389/fonc.2021.681429

**Published:** 2021-08-16

**Authors:** Zhengyu Yang, Ya Chen, Yanan Wang, Shuyuan Wang, Minjuan Hu, Bo Zhang, Baohui Han

**Affiliations:** Department of Pulmonary, Shanghai Chest Hospital, Shanghai Jiao Tong University, Shanghai, China

**Keywords:** epidermal growth factor receptor, tyrosine kinase inhibitors, next-generation sequencing, *TP53*, co-mutations

## Abstract

**Background:**

Co-mutations was associated with poor response to EGFR-TKIs. First-generation EGFR-TKIs combined with chemotherapy was reported to be more effective than TKIs alone in advanced lung adenocarcinoma patients.

**Objective:**

This retrospective study aimed to explore whether *EGFR*-mutant patients with co-mutations can benefit from EGFR-TKIs plus chemotherapy.

**Patients and Methods:**

We retrospectively collected data of 137 *EGFR*-mutant patients with advanced lung adenocarcinoma who underwent next-generation sequencing in our hospital in 2018. Among them, 96 were treated with EGFR–TKIs alone and 41 received EGFR–TKIs plus chemotherapy. We analyzed the progression-free survival (PFS) of patients with co-mutations using different treatments.

**Results:**

Concurrent *TP53* mutations, especially exon 4 and 6, were associated with a markedly shorter time to progression on EGFR-TKI monotherapy (11.4 months *vs*. 16.6 months, *P*=0.003), while EGFR–TKIs plus chemotherapy would benefit those patients more (with *TP53:* 11.4 months *vs*. 19.1 months, *P*=0.001, HR=0.407; without *TP53*: 16.6 months *vs*. 18.9 months, *P*=0.379, HR=0.706). The incidence of T790M after resistance was equal in patients treated with different treatments (53% *vs*. 53%, *P*=0.985).

**Conclusions:**

In our study, concurrent *TP53* mutations were found to be risk factors for EGFR-TKI monotherapy, but TKI combined with chemotherapy could eliminate this heterogeneity.

## Introduction

In lung adenocarcinoma (LAC), *Epidermal growth factor receptor* (*EGFR*) is one of the most common driver genes and can be detected in 40%-50% of Asian patients (1, 2). With the development of targeted therapy, most patients with *EGFR* mutations can benefit from first-generation EGFR tyrosine kinase inhibitors (EGFR-TKIs) (such as gefitinib, erlotinib, and icotinib, *etc*.) (3, 4). Several recent prospective studies have shown that *EGFR*-mutant patients using EGFR-TKIs combined with chemotherapy can have a better prognosis than TKI alone (5–7).

However, there is significant heterogeneity in patients’ objective responses to EGFR-TKI monotherapy, with about 20%-30% of patients failing to respond well or developing drug resistance in the early stage. Previous reports indicated that co-mutations may be associated with poor response to EGFR-TKIs (8–10). Therefore, we tried to explore whether patients with co-mutations can benefit from EGFR-TKIs plus chemotherapy.

In our research, we collected information on patients who used EGFR-TKIs plus chemotherapy and TKIs alone in our hospital. We used the data of next-generation sequencing (NGS) to analyze the most frequent co-mutations, and tried to provide some references for precise treatment.

## Materials And Methods

### Patients

**Figure 1:**
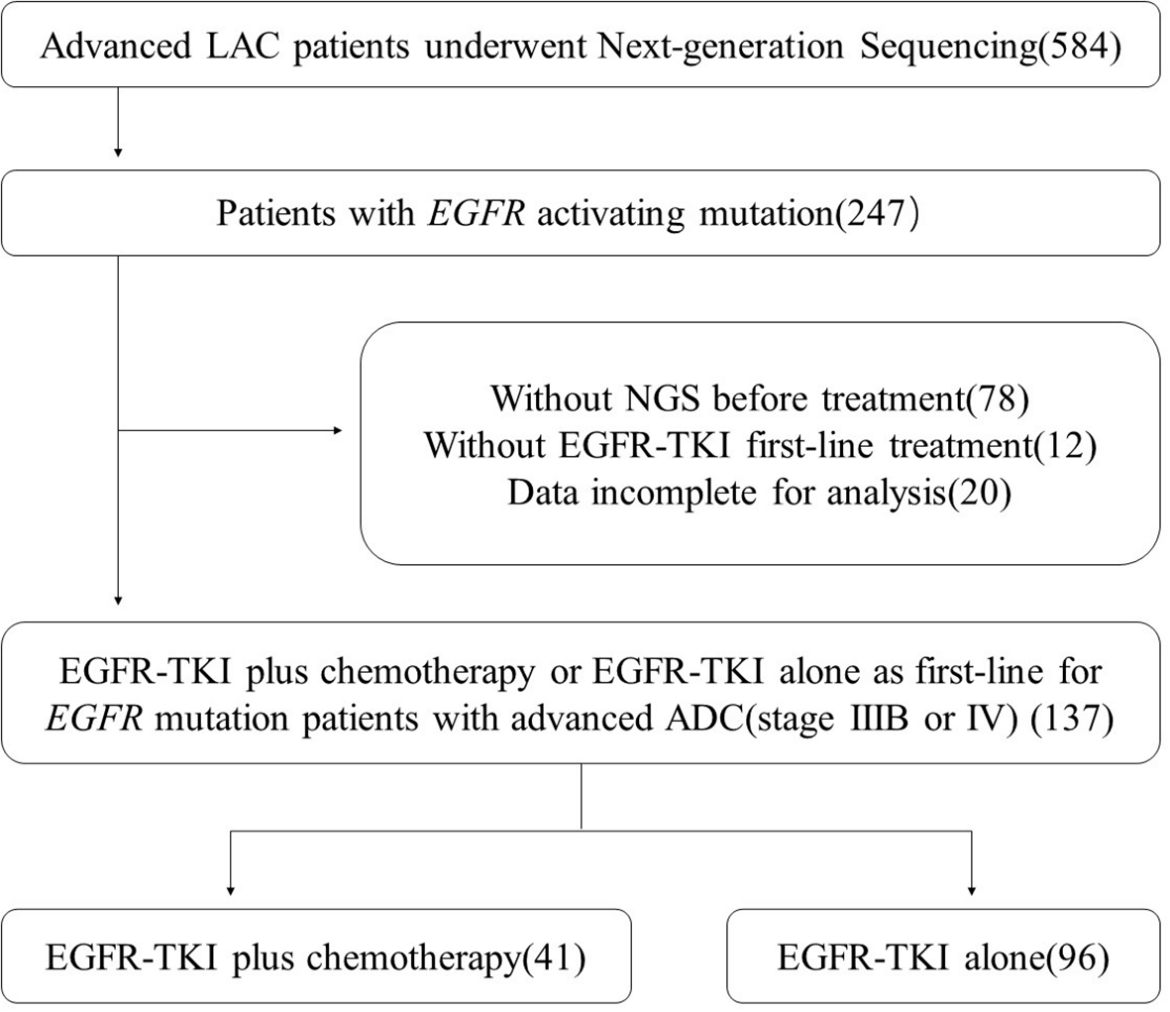
Patient selection flow-chart.

We collected LAC patients who underwent NGS in our hospital in 2018. The specific flow chart for screening patients is shown in **Figure 1**. We also collected the baseline characteristics of the enrolled patients, including age, gender, smoking status, TNM stage, ECOG-PS score, metastases status, and *EGFR* subtype.

At last, 137 patients were enrolled in our study and they met all the following screening criteria. First, they were diagnosed with advanced lung cancer (TNM stage IIIB or IV) and detected *EGFR* sensitive mutations (ex19 deletion or ex21 L858R mutation). Second, they underwent NGS before their first-line treatment and had complete follow-up data in our hospital. All patients gave informed consent before performing operation and treatment.

### Next-Generation Sequencing

**Figure 2:**
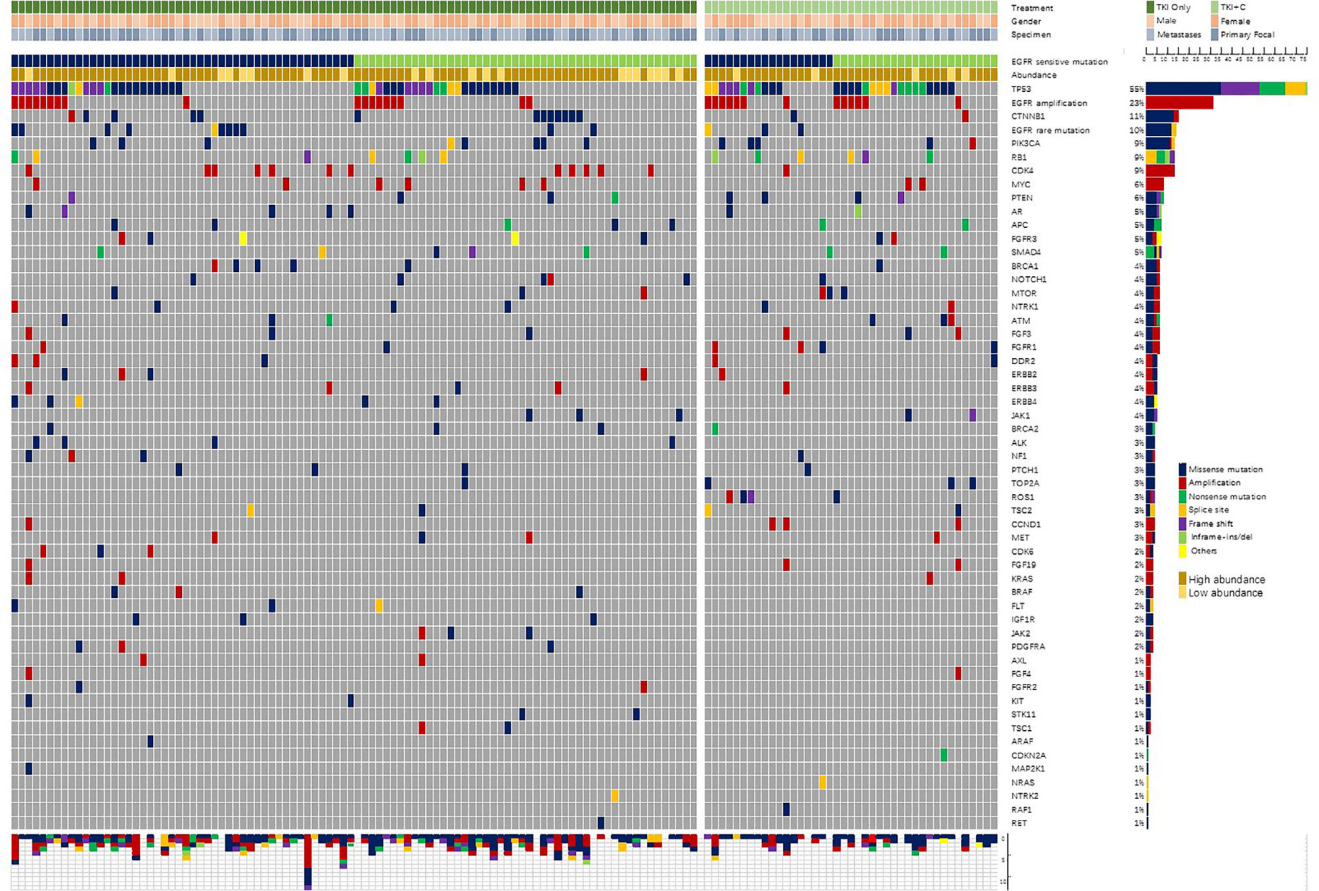
Oncoprint of genomic alterations identified in baseline tumor tissue (n = 137).

All surgically removed or biopsy tissues were fixed with formalin and embedded in paraffin. Tumor genomic DNA was extracted with the QIAamp DNA FFPE Tissue Kit (Qiagen, Hilden, Germany). As described previously (11), samples were sequenced by Nextseq500 sequencer (Illumina, Inc, San Diego, CA) and evaluated by a panel covering hotspot regions of 68 key cancer-related genes (**Supplementary Table 1**). The coverage depth of each sample could reach more than 1000×. The genetic profile of samples was shown in **Figure 2**.

According to previous reports (12), TP53 mutations were divided into 2 groups according to different exon mutation sites.

### Treatment and Follow-Up

The monotherapy group was administered first-generation EGFR-TKIs, and the specific dose was gefitinib 250 mg once a day, erlotinib 150 mg once a day, or icotinib 125 mg three times a day. The combination therapy group was given EGFR-TKIs combined with chemotherapy (mainly pemetrexed plus platinum) until the condition worsened or unacceptable toxicity occurred. The mean interval between consecutive chemotherapies was 4 weeks. Bases on the Response Evaluation Criteria in Solid Tumors (RECIST v1.1), patients were clinically evaluated every 4 to 6 weeks. Progression-free survival (PFS) is defined as the time from the initiating EGFR-TKIs to the occurrence of disease progression or the last follow-up (October 10, 2020). The median follow-up time was 24 months.

### Statistical Analysis

Chi-square test, Fisher’s exact test and Rank sum test were used to compare categorical variables and continuous variables between groups as appropriate. The Kaplan-Meier method and Log-rank tests were used for PFS analysis to compare the PFS of different groups. A *P* value of less than 0.05 was considered statistically significant. All analyses were performed on the Statistical Package for Social Science (SPSS, Chicago, IL version 22.0).

## Results

### Characteristics of Patients

A total of 584 advanced LAC patients underwent next-generation sequencing were included in the preliminary screening. The specific flow chart for screening patients is shown in **Figure 1**. Finally, 137 patients with advanced LAC (stage IIIB or IV) receiving EGFR-TKI plus chemotherapy or EGFR-TKI alone as first-line were included in our analysis.

Among the 137 patients, 96 (70%) were treated with EGFR–TKIs alone and 41 (30%) received EGFR–TKIs plus chemotherapy. In the combination therapy group, 39 patients were received pemetrexed plus platinum, and the other 2 patients with gemcitabine plus platinum. The average age in the monotherapy and combination therapy group was 61 years (42 to 80 years) and 62 years (35 to 87 years), respectively. There was no significant difference between the two groups in age, gender, smoking history, ECOG-PS, *EGFR* subtype, and metastasis status (**Table 1**). Among the specimens analyzed by NGS, 52% (71/137) were obtained from primary lung sites, while the others were from metastatic lymph node biopsy or pleural effusion embedding.

**Table 1:** Patients baseline characteristics.

	EGFR-TKI +chemotherapy (n = 41) (%)	EGFR-TKI (n = 96) (%)	*P* Value
Age, y, (range)	60.6 (42,80)	62.0 (35,87)	0.461
Sex			0.613
Female	22 (53.7)	56 (58.3)	
Male	19 (46.3)	40 (41.7)	
Smoking history			0.354
Yes	12 (29.3)	21 (21.9)	
No	29 (70.7)	75 (78.1)	
TNM stage			0.376
IIIB	2 (4.9)	11 (11.5)	
IV	39 (95.1)	85 (88.5)	
ECOG-PS			1.000
0-1	40 (97.6)	93 (96.9)	
2-3	1 (2.4)	3 (3.1)	
EGFR mutation			0.513
Exon 19	23 (56.1)	48 (50.0)	
Exon 21	18 (43.9)	48 (50.0)	
Brain metastasis	9 (22.0)	19 (19.8)	0.774
Bone metastasis	24 (58.5)	41 (42.7)	0.089

### Baseline Genomic Characteristics

In addition to *EGFR* sensitive mutations, a total of 364 individual cell mutations and functional mutations were found. At average, a single patient had 2.66 accompanying mutations. Patients with 21L858R mutation tended to have more concomitant mutations than patients with 19del mutation (2.89 *vs*. 2.44, *P*=0.183). The majority were missense mutations (47%, 171/364) and amplification (29%, 106/364). **Figure 2** showed the frequency and composition of the somatic mutations. *TP53* (55%, 75/137) was the most frequent concurrent mutation, followed by *EGFR* amplification (23%), *CTNNB1* (11%), *EGFR* rare mutations (10%), *PIK3CA* (9%), *RB1* (9%), *CDK4* (9%), *etc*. Twenty-five patients (18%) were identified with low-abundance *EGFR* mutations, which were detected in samples with a mutation frequency of less than 10%. What’s more, *EGFR* amplification (84%, 26/31), *RB1* (85%, 11/13) and *PTEN* (75%, 6/8) were often accompanied by *TP53* mutations.

In our cohort, TP53 mutation sites were distributed in exons 3-10. Of the 75 patients with TP53 mutations, 1, 6, 24, 14, 15, 9, 5, 1 were located in each exon, respectively.

### Outcomes in Monotherapy Group and Combination Therapy Group

After monotherapy or combination therapy, the ORR (the proportion of patients with a confirmed complete or partial response) were 53.1% and 73.2%, respectively(*P*=0.029). The disease control rate (the proportion of patients with a confirmed complete or partial response or stable disease) were 90.6% and 97.6%, respectively(*P*=0.284). Of the 28 patients with brain metastases at baseline, excluding 8 patients who received local therapy, the intracranial ORR were 72.7% and 77.7%, respectively (*P*=1.000).

**Figure 3:**
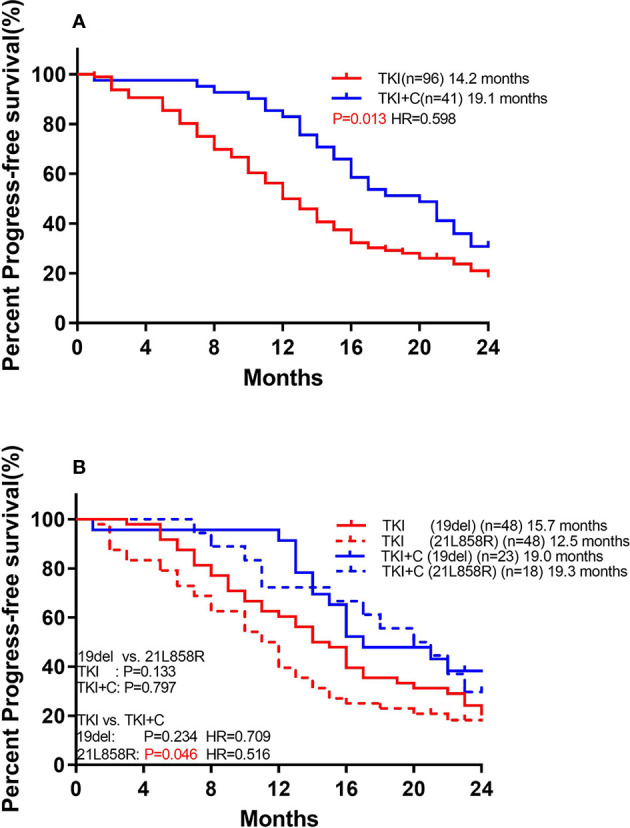
**(A)** Comparison of monotherapy and combination therapy for PFS in patients with *EGFR* mutation. **(B)** Comparison of association of *EGFR* subtype with PFS to monotherapy and combination therapy in patients with *EGFR* mutation.

The patients who received combination therapy had significantly longer PFS than those who received monotherapy (**Figure 3A**; 19.1 months *vs*. 14.2 months, *P*=0.018, HR=0.598 95%Cl, 0.391-0.914). Compared with patients with *EGFR* 19del, patients with *EGFR* 21L858R tended to have shorter PFS in monotherapy group (**Figure 3B**; 12.5 months *vs*. 15.7 months, *P*=0.133), whereas they benefited more from combination therapy (19del: 19.0 months *vs*. 15.7 months, *P*=0.234, HR=0.709; 21L858R: 19.3 months *vs*. 12.5 months, *P*=0.046, HR=0.516).

We performed univariate and multivariate analyses of baseline characteristics and high-frequency mutations (including *TP53*, *EGFR* amplification, *CTNNB1*, *EGFR* rare mutations, *PIK3CA*, *RB1* and *CDK4*) in patients treated with monotherapy (**Table 2**). We found that concurrent *TP53* mutations (HR: 2.044, 95%Cl, 1.295 to 3.225, *P*=0.002) had a significant effect on PFS in both analyses, while *EGFR* amplification only had a negative effect in univariate analysis (HR: 1.852, 95%Cl, 1.061 to 3.231, *P*=0.030).

**Table 2:** Univariate and multivariate analysis for PFS in monotherapy group.

PFS				
Characteristics	Univariate analysis HR (95%Cl)	*P*	Multivariate analysis HR (95%Cl)	*P*
Age				
≤60 Yr *vs*. >60 Yr	0.859 (0.544 – 1.358)	0.515		0.831
Gender				
Male *vs*. female	0.916 (0.583 – 1.439)	0.703		0.498
Smoking Status				
Yes *vs*. no	1.498 (0.880 – 2.550)	0.136		0.144
TNM Stage				
IIIB *vs*. IV	0.877 (0.403 – 1.908)	0.741		0.423
*EGFR* sensitive mutation				
19DEL *vs*. 21L858R	0.846 (0.675 – 1.060)	0.147		0.072
*EGFR* mutation abundance				
Low *vs*. high	1.445 (0.807 – 2.584)	0.215		0.226
*EGFR* amplification				
With *vs*. without	1.852 (1.061 – 3.231)	0.030		0.271
*TP53*				
With *vs*. without	1.933 (1.226 – 3.048)	0.005	2.044 (1.295 – 3.225)	0.002
*CTNNB1*				
With *vs*. without	1.013 (0.545 – 1.885)	0.967		0.372
*EGFR* rare mutations				
With *vs*. without	0.716 (0.342 – 1.499)	0.376		0.434
*PIK3CA1*				
With *vs*. without	0.894 (0.388 – 2.060)	0.792		0.828
*RB1*				
With *vs*. without	2.017 (0.918 – 4.429)	0.080		0.273
*CDK4*				
With *vs*. without	0.942 (0.469 – 1.892)	0.867		0.389

**Figure 4:**
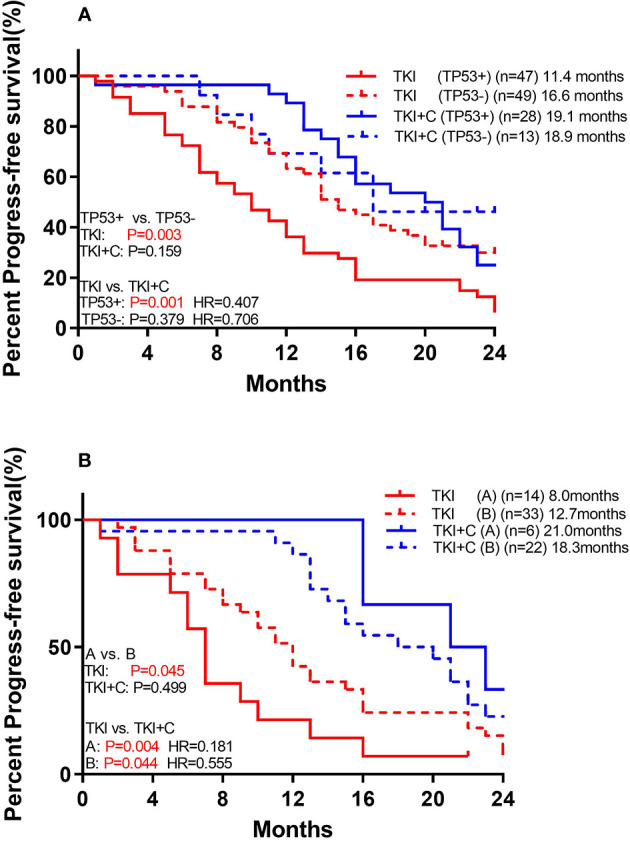
**(A)** Comparison of association of concurrent *TP53* mutation with PFS to monotherapy and combination therapy in patients with *EGFR* mutation **(B)** Comparison of association of *TP53* subtypes with PFS to monotherapy and combination therapy in patients with *EGFR* mutation. [Group **(A)** included exon 4 and 6. Group **(B)** included the remaining mutant types].

We also compared the outcomes of patients with and without *TP53* mutations. Of the 96 patients receiving monotherapy, those with concomitant *TP53* mutation showed a significantly worse response (**Figure 4A**; 11.4 months *vs*. 16.6 months, *P*=0.003). However, patients with or without *TP53* yielded equivalent PFS in combination therapy group (18.9 months *vs*. 19.1 months, *P*=0.552). Patients with *TP53* benefited more from combination therapy (with *TP53*: 11.4 months *vs*. 19.1 months, *P*=0.001, HR=0.407; without *TP53*: 16.6 months *vs*. 18.9 months, *P*=0.379, HR=0.706).

TP53 mutations were further divided into two groups. Group A included exon 4 and 6, and both of these two types of mutations had poor prognosis. Group B included the remaining mutant types which shared a good prognosis. Among monotherapy patients, PFS was significantly lower in group A than in group B (**Figure 4B**; 8.0 months *vs*. 12.7 months, P=0.045). Both groups benefited from the combination therapy, but group A benefited more (Group A: 8.0 months *vs*. 21.0 months, *P*=0.004, HR=0.181; Group B: 12.7 months *vs*. 18.3 months, *P*=0.044, HR=0.555).

### Resistance Mechanisms in Monotherapy Group and Combination Therapy Group

Then, we analyzed the baseline and post-resistance samples to explore the resistance mechanisms associated with different treatments. After first-line treatment progression, in patients undergoing *EGFR* T790M testing, 53% (20/38) in monotherapy group and 53% (11/21) in combination therapy group were positive, which was pretty equivalent (*P*=0.985). Mechanisms related to resistance in 26 patients who underwent NGS again after progression were summarized in **Table 3**. Among them, the emergence of *MET* amplification at PD occurred in 3 of monotherapy group and 1 of combination therapy group. One *ERBB2* amplification patient was observed in both groups.

**Table 3:** Resistance mechanism to monotherapy and combination therapy.

	Monotherapy (n = 13)	Combination therapy (n = 13)
**T790M**	4 (31%)	6 (46%)
**T790M+ *ERBB2* amp**	1 (8%)	
***MET* amp**	3 (23%)	1 (8%)
***ERBB2* amp**		1 (8%)
***RB1***	1 (8%)	
***RB1*+ *TP53***		1 (8%)
***FGFR2***	1 (8%)	
***PTEN***	1 (8%)	
***BRAF***		1 (8%)
***MYC* amp**		1 (8%)
**Unknown**	2 (15%)	2 (15%)

## Discussion

In our study, we retrospectively analyzed the concomitant genomic alterations of advanced LAC patients with *EGFR* mutations and accessed the clinical efficacy of EGFR-TKI plus chemotherapy as first-line treatment.

We found that *EGFR* mutations were frequently associated with other mutations, with an average of 2.66 accompanying mutations, consistent with previous reports (13). The most common accompanying mutations were *TP53* (55%), *EGFR* amplification (23%), *CTNNB1* (11%), *EGFR* rare mutations (10%), *PIK3CA* (9%), *RB1* (9%), *CDK4* (9%), and so on. Previous studies have found that co-mutations may activate the alternative signaling pathway or increase tumor heterogeneity, thereby affecting the efficacy of EGFR-TKIs (9, 14).

We found that *TP53* mutations, especially exon 4 and 6, were associated with a markedly shorter time to progression on EGFR-TKI monotherapy, which was consistent with previous reports (8–10, 12). *TP53* is a key tumor suppressor gene that can enhance sensitivity to EGFR-TKIs and radiotherapy by inducing cell-cycle arrest, apoptosis, and repair of DNA damage (15). The complete loss of *TP53* function, mainly manifested as single-base substitution and loss of alleles, can catalyze the transformation potential of oncogene drivers in lung cancer and inhibit tumor response to chemotherapy, radiotherapy and EGFR-TKIs (15, 16). However, in the combination therapy group, patients with *TP53* also showed a good response, and there was no significant difference in PFS compared with patients without *TP53*. This means that the combination of EGFR-TKI and chemotherapy will benefit patients with concurrent *TP53* mutations more.

Several previous studies reported that *EGFR* amplification in *EGFR*-mutant patients was associated with a longer PFS in TKI treatment (17, 18). In our study, *EGFR* amplification was a risk factor for PFS in univariate analysis but not in multivariate analysis, possibly because it was mainly accompanied by TP53.

In addition, many studies have shown that patients with *EGFR* 21L858R mutation do not respond as well to EGFR-TKI as patients with *EGFR* 19del mutation (19, 20). This may be attributed to the different intrinsic sensitivity of the two mutations to EGFR-TKIs (21). This trend was also observed in our study, in which patients with 21L858R had shorter PFS than those with 19del, but they benefited more from combination therapy.

EGFR-TKI combined with chemotherapy has been reported in a large number of prospective studies to delay resistance (5–7). This combination therapy was found to induce cell apoptosis and inhibit Akt and extracellular signal-regulated kinase phosphorylation (22), and EGFR-TKIs could reduce the level of thymidine synthase to improve the efficacy of pemetrexate (23). What’s more, the proportions of patients with T790M positive after progression were similar in the combination therapy and monotherapy group, which meant that the majority of patients with the first-line combination therapy could be successfully treated with the sequential therapy of osimertinib. However, in addition to excellent effect, clinically relevant grade ≥ 3 toxicity in the combination therapy group were doubled (5, 6). There may be more patients over the age of 75 with a high ECOG-PS score in the clinical course, so we need to identify patients who would benefit more from the combination therapy. In our study, we found that patients with 21L858R or coexisting *TP53* mutations did not respond well to monotherapy, but benefited more from combination therapy. In addition, FLAURA (NCT02296125) showed osimertinib as first-line treatment yielded more benefits than first-generation EGFR-TKIs, providing an alternative option for patients with EGFR mutations (24, 25).

Our study has the following limitations. First, as a retrospective study, we failed to compare the adverse effects of different treatments due to incomplete records. Second, although there was no significant difference in baseline characteristics among patients receiving different treatments, we recognized the existence of selection bias that patients with comorbidities were more likely to be recommended for monotherapy. Third, the mechanism by which combination therapy benefits patients with *TP53* mutations remains unclear and needs further study.

In summary, we retrospectively analyzed genomic changes in patients with advanced lung adenocarcinoma with sensitizing *EGFR* mutations and found that *TP53* was the most frequent concurrent mutations. Grouped by next-generation sequencing results, we compared the efficacy of monotherapy versus combination therapy. We found that patients with 21L858R mutation or concurrent *TP53* mutations did not respond well to EGFR-TKIs alone, but benefited more from EGFR-TKIs plus chemotherapy. In the future clinical treatment process, we should consider to stratify patients according to their *EGFR* subtype and concurrent mutations, and develop more targeted treatment programs.

## Data Availability Statement

The original contributions presented in the study are included in the article/**Supplementary Material**, further inquiries can be directed to the corresponding authors.

## Ethics Statement

The studies involving human participants were reviewed and approved by The institutional review board of Shanghai Chest Hospital. The patients/participants provided their written informed consent to participate in this study.

## Author Contributions

ZY, YC, and YW both have substantial contributions to the conception or design of the work, the collection and analysis of data, the writing and edit of the article. The rest authors have given substantial contributions to the work by providing editing and writing assistance. All authors contributed to the article and approved the submitted version.

## Funding

This work was supported by the foundation of Shanghai Chest Hospital (Project No. YJXT20190102); the program of system biomedicine innovation center from Shanghai Jiao Tong University (Project No. 15ZH4009); Shanghai Jiao Tong University School of Medicine (Project No. 15ZH1008) and the foundation of Chinese society of clinical oncology (Project No. Y2019AZZD-0355).

## Conflict of Interest

The authors declare that the research was conducted in the absence of any commercial or financial relationships that could be construed as a potential conflict of interest.

## Publisher’s Note

All claims expressed in this article are solely those of the authors and do not necessarily represent those of their affiliated organizations, or those of the publisher, the editors and the reviewers. Any product that may be evaluated in this article, or claim that may be made by its manufacturer, is not guaranteed or endorsed by the publisher.

## Supplementary Material

Supplementary Table 1 Sixty-eight cancer-related Genes sequenced in our panel.
