# Supplementary material for: Efficacy of EGFR-TKI Plus Chemotherapy or Monotherapy as First-Line Treatment for Advanced EGFR-Mutant Lung Adenocarcinoma Patients With Co-Mutations
Source: Front Oncol. 2021 Aug 16;11:681429. doi: 10.3389/fonc.2021.681429 (PMC8415777; doi:10.3389/fonc.2021.681429)
Supplement: Supplementary Table 1 — Sixty-eight cancer-related Genes sequenced in our panel. [file Table_1.docx]

**Supplemental Table 1**. Sixty-eight Cancer-related Genes Sequenced in Our Panel.

| Gene Symbol | Target Region |
| --- | --- |
| AKT1 | Whole exons |
| ALK | Whole exons plus intron 19 |
| APC | Whole exons |
| AR | Whole exons |
| ARAF | Whole exons |
| ATM | Whole exons |
| AXL | Whole exons |
| BCL2L11 | Intron 2 |
| BRAF | Whole exons |
| BRCA1 | Whole exons |
| BRCA2 | Whole exons |
| CCND1 | Whole exons |
| CD74 | Whole exons |
| CDK4 | Whole exons |
| CDK6 | Whole exons |
| CDKN2A | Whole exons |
| CTNNB1 | Whole exons |
| CYP2C19 | rs4244285; rs4986893 |
| CYP2D6 | rs1065852; rs1135840; rs1058164 |
| CYP3A4 | rs55951658; rs4985908; rs28371759 |
| DDR2 | Whole exons |
| DPYD | rs3918290; rs1801159 |
| EGFR | Whole exons |
| ERBB2 | Whole exons |
| ERBB3 | Whole exons |
| ERBB4 | Whole exons |
| ESR1 | Whole exons |
| FGF19 | Whole exons |
| FGF3 | Whole exons |
| FGF4 | Whole exons |
| FGFR1 | Whole exons |
| FGFR2 | Whole exons |
| FGFR3 | Whole exons plus intron 17-19 |
| FLT3 | Whole exons |
| HRAS | Whole exons |
| IDH1 | Whole exons |
| IDH2 | Whole exons |
| IGF1R | Whole exons |
| JAK1 | Whole exons |
| JAK2 | Whole exons |
| KDR | Whole exons |
| KIT | Whole exons |
| KARS | Whole exons |
| MAP2K1 | Whole exons |
| MET | Whole exons |
| MTOR | Whole exons |
| MYC | Whole exons |
| NF1 | Whole exons |
| NOTCH1 | Whole exons |
| NRAS | Whole exons |
| NTRK1 | Whole exons plus intron 9 |
| NTRK2 | Whole exons |
| NTRK3 | Whole exons |
| PDGFRA | Whole exons |
| PIK3CA | Whole exons |
| PTCH1 | Whole exons |
| PTEN | Whole exons |
| RAF1 | Whole exons |
| RB1 | Whole exons |
| RET | Whole exons plus intron 11 |
| ROS1 | Whole exons plus intron 31-35 |
| SMAD4 | Whole exons |
| SMO | Whole exons |
| SKT11 | Whole exons |
| TOP2A | Whole exons |
| TP53 | Whole exons |
| TSC1 | Whole exons |
| TSC2 | Whole exons |
